# Motor-cognitive dual-task performance: effects of a concurrent motor task on distinct components of visual processing capacity

**DOI:** 10.1007/s00426-017-0951-x

**Published:** 2017-12-01

**Authors:** E. C. S. Künstler, K. Finke, A. Günther, C. Klingner, O. Witte, P. Bublak

**Affiliations:** Hans Berger Department of Neurology, Jena University Hospital, Am Klinikum 1, 07747 Jena, Germany

## Abstract

Dual tasking, or the simultaneous execution of two continuous tasks, is frequently associated with a performance decline that can be explained within a capacity sharing framework. In this study, we assessed the effects of a concurrent motor task on the efficiency of visual information uptake based on the ‘theory of visual attention’ (TVA). TVA provides parameter estimates reflecting distinct components of visual processing capacity: perceptual threshold, visual processing speed, and visual short-term memory (VSTM) storage capacity. Moreover, goodness-of-fit values and bootstrapping estimates were derived to test whether the TVA-model is validly applicable also under dual task conditions, and whether the robustness of parameter estimates is comparable in single- and dual-task conditions. 24 subjects of middle to higher age performed a continuous tapping task, and a visual processing task (whole report of briefly presented letter arrays) under both single- and dual-task conditions. Results suggest a decline of both visual processing capacity and VSTM storage capacity under dual-task conditions, while the perceptual threshold remained unaffected by a concurrent motor task. In addition, goodness-of-fit values and bootstrapping estimates support the notion that participants processed the visual task in a qualitatively comparable, although quantitatively less efficient way under dual-task conditions. The results support a capacity sharing account of motor-cognitive dual tasking and suggest that even performing a relatively simple motor task relies on central attentional capacity that is necessary for efficient visual information uptake.

## Introduction

If we allocate undivided attention to a task, its execution will often be more successful as compared to situations when our attention is distracted by a concurrent task. Thus, it is everyday experience that paying attention to the visual environment is affected by the concurrent execution of a motor task. Consider driving a car whilst repeatedly pressing the buttons of your car stereo device in search of your favourite radio program or CD track. In such a situation, your monitoring of the traffic events outside will likely be rendered less efficient compared to a condition when you are focussed on the visual task alone. Empirical data corroborate this view. For example, Mioni et al. (2016) found temporal discrimination thresholds in the visual but not the auditory modality to be elevated by performing a concurrent finger tapping task in young healthy subjects. Similarly, Fuller and Jahanshahi (1999) reported that, in patients with schizophrenia, the performance of a task requiring visual-selective attention declined during concurrent finger tapping. These data suggest that even relatively simple motor tasks can significantly affect the efficiency of visual processing.

One approach to understand the performance decline typically observed under dual-task conditions, when two continuous tasks have to be executed simultaneously, is a resource sharing account (see Tombu & Jolicoeur, 2004, for an overview). This framework assumes that two tasks can be performed in parallel, but that the amount of processing capacity is strictly limited. Due to the limited resources, the available processing capacity has to be shared between the two tasks, rendering task processing of both tasks less efficient. The decrease of processing efficiency under dual-task conditions, compared to the processing of each single task in isolation, is observed as the dual-task cost. Several versions of the resource sharing model have been proposed. Kahneman’s (1973) original proposal suggested a more or less undifferentiated pool of mental resources that can be allocated to different task demands. Navon (1984), and Wickens (2002) assumed multiple resources that can be shared across tasks, giving rise to dual-task costs whenever two or more task processes or stages draw from the same specific resource. A special case are central capacity sharing models (Navon & Miller, 2002; Tombu & Jolicoeur, 2004) which accept the idea of multiple task stages, but assume resource sharing at central processing stages only. These models consider the structural bottleneck account of dual-task costs, with its implication of serial task processing at central stages (Pashler, 1994), as a special case of capacity sharing, when task 1 and task 2 get all of the available capacity, respectively, in serial succession. A model that encompasses aspects of both the structural bottleneck, and of the resource sharing account, has been proposed by Logan and Gordon (2001) in their ‘executive control of the theory of visual attention’ (ECTVA) model.

The ‘theory of visual attention’ (TVA) introduced by Bundesen (1990; see also Bundesen, Vangkilde, & Petersen, 2015, for a recent update) is a framework well suited for assessing how the efficiency of visual information uptake is affected by a concurrent motor task. TVA conceptualizes visual processing capacity as a set of attentional parameters. These parameters can be estimated, on the individual level, by modelling a subject’s performance in a simple psychophysical task, i.e., whole report of briefly presented letter arrays. In short, TVA assumes that visual information uptake is accomplished across two processing waves. During the first, unselective wave, evidence values are computed during a massive parallel processing of the visual input, where objects from the display are matched to long-term memory representations. In the second, selective wave of processing, the available attentional capacity is distributed across the objects in the visual field, and weighted according to the evidence values. All objects compete with each other in a race towards visual short-term memory (VSTM) which has a limited storage capacity of about four elements in healthy, young, participants. Objects receiving more attentional weight race with a faster speed and gain higher probability to be encoded into VSTM. Encoded objects are selected and available for further processing in the cognitive system. Thus, in TVA, the efficiency of visual information uptake is represented by three parameters reflecting the perceptual threshold (parameter *t*0), the rate of visual processing (parameter C), and the storage capacity of VSTM (parameter K). These parameters reflect origin, slope, and asymptote, respectively, of the exponential growth function by which the individual whole report performance is modelled according to the equations provided by TVA (see Kyllingsbæk, 2006; Habekost, 2015, for a tutorial overview).

Based on TVA, it is possible to individually describe attentional parameters representing the efficiency of visual information processing. Compared to ‘classical’ response time based measures, a number of important advantages arise with respect to the analysis of dual-task effects. It is not only possible to assess the effects induced by a concurrent motor task on visual information uptake by quantifying, for each individual participant, whether and to what degree changes of the perceptual threshold, rate of information uptake, and VSTM storage capacity are invoked. In addition, TVA-based analysis also allows for a comparison between single- and dual-task conditions according to qualitative aspects related to task processing. In TVA, it is assumed that the parameter visual processing speed *C* and VSTM storage capacity *K* are indexing processes that are relatively constant, within a given individual, across comparable stimulus and task conditions. Indeed, they were interpreted as having a latent trait character (e.g., Finke et al., 2012). However, it might be possible that, when measured in a dual-task scenario, these parameters reflect variable performance from moment to-moment, traded off in a time-sharing manner. In other words, in the dual-task condition, participants might start and stop the entire task process in which the TVA parameter estimates are embedded, depending on whether or not the participants were paying attention to the visual task. Then, the *C* estimate, for example, rather than reflecting a constant rate of information uptake across the dual-task condition, might be an average of actual *C* and a non-operating task (where *C* could possibly even equal 0). Therefore, two statistical analyses were run to explore whether, in dual-task conditions, the TVA parameter estimates actually reflect a relatively constant performance that can be validly modelled using the TVA-fitting process, or rather provide an overall average across very low versus optimal performance. First, goodness of fit measures were obtained for each participant that reflect the degree to which variance in the empirical performance in the different whole report conditions can be predicted by the individual TVA parameter estimates. Second, the variability of the individual parameter estimates under single- and dual-task conditions was assessed by a bootstrapping procedure (Efron & Tibshirani, 1993) to investigate the possibility of a broader distribution of the estimates under dual-task conditions.

Effects of a concurrent visual task have been recently assessed within a TVA-based framework by Poth, Petersen, Bundesen, & Schneider, (2014). These authors found a reduction of visual processing speed, but no effects on the perceptual threshold and the storage capacity of the VSTM. Our study combines—to the best of our knowledge, for the first time—the TVA approach with a continuous motor task in a dual-task procedure. We assessed whether visual processing speed is also affected under a concurrent non-visual task, and whether VSTM storage capacity would be affected as well. As part of this special issue, this attempt can offer new insight into how visual processing is affected by performance of a concurrent motor task. It also offers novel possibilities to assess qualitative differences between single- and dual-task conditions.

## Methods

### Participants

A total of 24 right-handed participants (10 female), aged between 40 and 71 years (*M* = 57.0; SD = 9.5), took part in this study. All were right-handed (verified by the Edinburgh Handedness Inventory; EHI; Oldfield, 1971) and had normal or corrected-to-normal vision. On average, they received *M* = 11.5 years of education (SD = 1.8), and had an IQ of *M* = 107.1 (SD = 9.9), as estimated by a German vocabulary test (MWT-B; Lehrl, 1999). All participants were without any history of neurological or psychiatric disease. The study was approved by the Ethics Committee of the Jena University Hospital, and all participants gave written informed consent prior to participation, in accordance with the Declaration of Helsinki. Each participant received a reimbursement of €30.

### Procedure

Participants underwent a single session which lasted approximately 2 h, with 40 min used for questionnaires and screening tests, and the remaining time allotted to the experimental conditions, with breaks being taken as needed.

### Tapping task

The tapping task used a simple sequence which consisted of using the index finger of the dominant hand to press the “1” key, and the middle finger of this same hand to press the “2” key on a separate numeric keyboard. This “1, 2” sequence was then tapped repetitively at a subjectively preferred speed. As all participants were right handed, the sequence tapped was the same for each participant. Following the methodology described by Kane and Engle (2000), this tapping task consisted of three blocks: the first block, which lasted 30 s, familiarised the participant with the sequence. If poorly performed, this block could be repeated. If successfully executed, the second block commenced, during which the average tapping speed was calculated over a duration of 60 s. If the wrong key was pressed, auditory feedback in the form of a beep was provided. If this block was also successfully completed, the participant could then go on to the final block. Here, the average tapping speed calculated in the second block was added to a tolerance buffer of 150 ms and was used as the cut-off speed for the participant’s subsequent performance. If the participant was too slow by taking longer to press a key than the time stipulated by this average tapping speed, or pressed the wrong key, auditory feedback was again provided. This final block lasted for 3 min. This time-span was chosen as 3 min reflects the average length of a block in the whole report task. All participants were asked whether they could tap without any discomfort for this period and none of them experienced any problems. Each tap made by the participant in this final block was recorded in a text file, along with the time stamp of when the tap was made, which key was pressed, the correct response, and how long it took for the key to be pressed. This allowed for error rates and tapping speeds to be established for each participant post hoc, as well as allowing for a comparison between the time stamps of each response on each task to be made.

### Whole report task

**Fig. 1:**
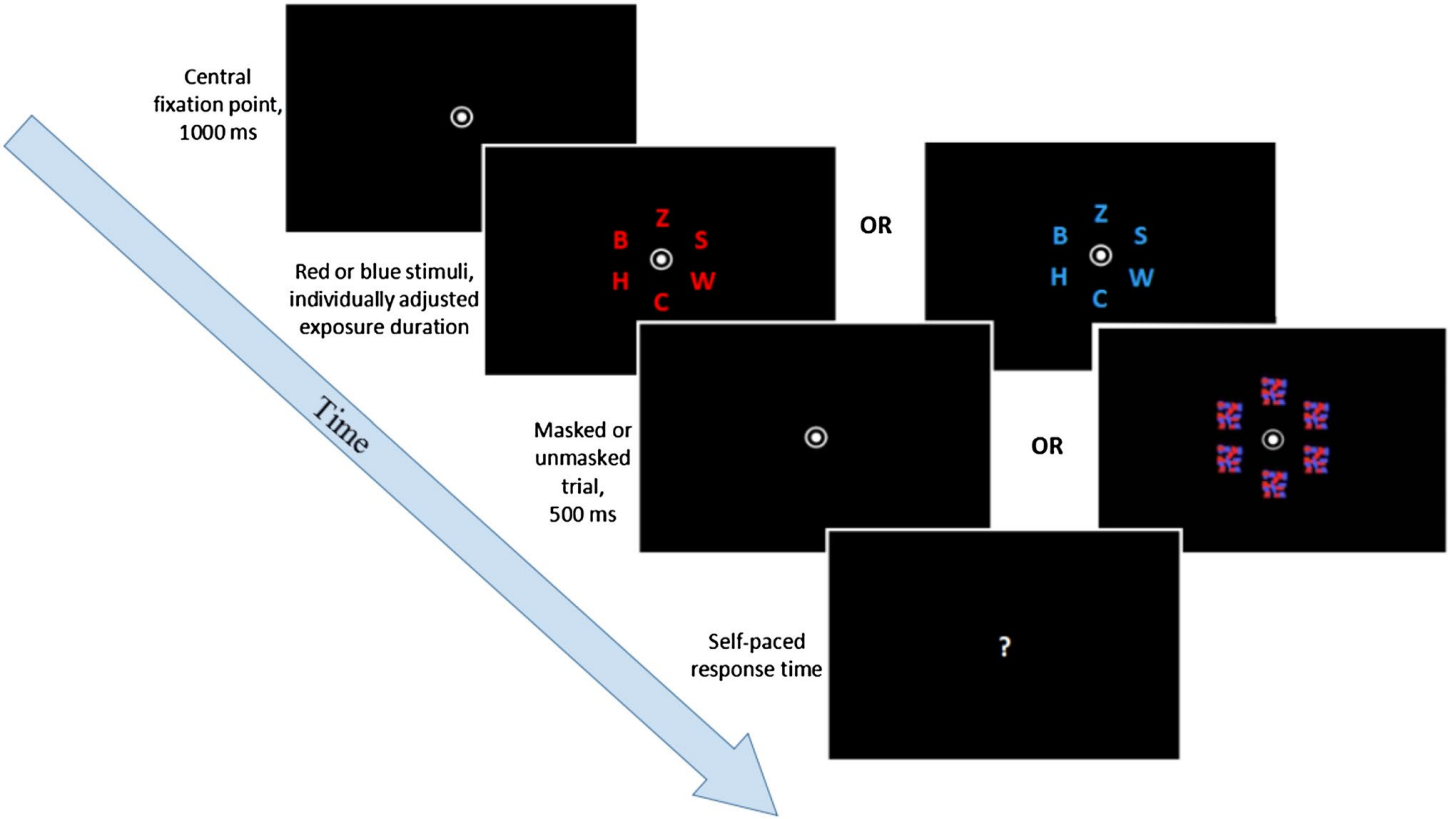
Whole report trial sequence

The whole report task was run using Matlab (MathWorks, 2012), using Psychtoolbox (Brainard, 1997). Participants received task instructions on-screen, along with two examples to elucidate the instructions. Following this, a pre-test consisting of 12 triples of trials divided into 4 blocks, with 12 trials per block, was run. This pre-test familiarised the participant with the task, and identified the appropriate exposure durations for each participant using an adaptive staircase model. Each triple consisted of two trials that were not used for adjustment. These were either unmasked with exposure duration of 200 ms or masked with exposure duration of 250 ms. One trial in each triple was critical for adjustment; this was masked and initially displayed for 100 ms. If at least one letter in such a critical trial was reported correctly, the exposure duration was decreased by 10 ms in the following critical trial. This was repeated until a final exposure duration was identified at which the participant could not even report one letter correctly. This exposure duration was determined as the lowest exposure duration and was combined with four longer exposure durations during the remainder of the experiment, which were picked from a pre-defined list based on the value of the lowest exposure duration. In 18 participants, the exposure durations used were 10, 20, 40, 90, and 200 ms. A further three participants had exposure durations of 20, 40, 60, 120, and 210 ms, whilst one participant had exposure durations of 30, 50, 80, 130, and 220 ms. Finally, two participants were tested using exposure durations of 40, 60, 100, 150, and 230 ms. In five unmasked conditions, stimuli were followed by a mask, to avoid visual persistence effects. The mask consisted of red-and-blue scattered squares of 1.3° size appearing on each stimulus location for 500 ms. Furthermore, to enhance variability of exposure durations, two unmasked conditions were additionally used, i.e., the second shortest and the longest exposure durations were presented both masked and unmasked. In unmasked trials, visual persistence increases the duration of information uptake by several hundred milliseconds (Sperling, 1960; Dick, 1974). This duration is estimated by parameter *µ* in TVA-based fitting of whole report performance, a parameter which only serves the valid estimation of the remaining parameters here, and is of no additional interest for this study. This resulted in seven effective exposure conditions, with each condition having 20 trials. The whole experiment thus consisted of 140 trials, which were divided into 4 blocks. Such exposure duration variability allowed measuring a broad range of whole report performance. Lower exposure durations allow valid estimations of the perceptual threshold *t*0 at lower exposure durations, which is also decisive for that of the rate of information uptake in ms at *t*0, i.e., for estimating visual processing speed *C*. Higher exposure durations are necessary for receiving precise estimates of the asymptote level of performance, i.e., of VSTM storage capacity *K*. An example of a trial sequence is given in Fig. 1.

As can be seen from Fig. 1, a fixation point was presented on the screen for a duration of 1000 ms. Following this, six different isoluminant letters were presented equidistantly in a circle around the fixation point. These target letters were either all red or all blue [CIE red = (0.49, 0.515, 0.322), CIE blue = (0.49, 0.148, 0.068)], and were selected randomly from a pre-specified set of letters (excluding the letters *I, Q*, and *Y*). The size of these letters was 1.5 cm by 1.5 cm, with the luminance being set to 0.49 cd/m^2^, thereby ensuring that both red and blue targets had the same level of task difficulty. In masked trials, the masks consisted of 2.0 cm by 2.0 cm squares of overlapping blue [Colour space: CIE *L* × *a* × *b* blue = (17.95; 45.15; − 67.08)] and red [CIE *L* × *a* × *b* red = (28.51; 46.06; 41.28)] flecks. After this, the screen went blank, and at this point, the participant had to verbally report as many target letters as possible, in any order. It was emphasised that this was not a speeded task, thereby allowing each participant to take as much time as necessary in making the responses. The researcher, who was seated to the side and slightly behind the participant, then entered the reported letters via a keyboard before proceeding to the next trial. The reported letters, as well as the time-stamps of each trial, were exported to a text file. After each block, participants received visual, on-screen feedback as to their accuracy on the letters they actually reported. In order to avoid both too liberal and too conservative responses, participants were encouraged to aim for an accuracy rate of 70–90%, indicated by a green area on the accuracy bar. If their accuracy was below 70%, participants were asked to only report those letters they were fairly confident of having seen. If the accuracy was over 90%, participants were encouraged to be less conservative by reporting more target letters, even if they did not feel entirely confident.

### Dual-task

The task order was counterbalanced, with 12 participants completing the single-task condition before the dual-task condition, and 12 participants completing it afterwards. In the dual-task, all participants started with the training and speed adjustment blocks of the tapping task before the whole report paradigm was subsequently started. During the dual-task, it was ensured that the participants did not visually monitor their tapping on the keyboard, but instead constantly fixated on the screen. This screen was adjusted for each participant, such that the central fixation point was at eye level. Due to the set-up of the apparatus, participants’ hands were located below the periphery of their visual field. Thus, to visually monitor their tapping, they would have had to move their heads to be able to see their hands (a mere shifting of the gaze downwards would not have been sufficient). The experimenter specifically monitored this, and ensured that no participant looked away from the central fixation point throughout the dual-task condition.

### Parameter estimation

Data obtained through the whole report paradigm were analysed using the LIBTVA script developed by Dyrholm (2012) and run through Matlab (MathWorks, 2012) to obtain a TVA-based maximum likelihood fit for the data of each participant. This fitting method uses the observed data points to extrapolate the probabilistic parameters, utilising the fixed-capacity independent race model (see Shibuya & Bundesen, 1988). Moreover, to assess the data in which both tasks were successfully executed, dual-task trials in which a tapping error had occurred were excluded from the analysis. This yielded information regarding the goodness of fit, and the various visual attentional parameters of each participant, and how they were affected by motor-cognitive dual-tasking.

In addition to the exact parameter estimates, 200 bootstrapping estimates were derived (Efron & Tibshirani, 1993) to obtain quantitative estimates of the robustness of the maximum likelihood estimates produced by the TVA fitting (see Habekost & Bundesen, 2003). To that end, the original dataset was resampled by drawing 140 “new” trials, at random, with replacement, from the original sample of 140 trials. The algorithm was repeated 200 times (for each experimental condition and participant) and a TVA-based maximum likelihood fit was computed for each of the resulting 200 bootstrapping samples. The standard estimates of these bootstrapping estimates may be taken as quantitative estimates of the standard errors of the original parameter estimations (Habekost & Bundesen, 2003). Note that, as during resampling, each original trial can be drawn 0, 1, 2, …, or up to n times, resampling an original mixture of trials with fluctuating, “normal” and “0” rates, of information should result in increased standard errors of the bootstrapping estimates. On the other hand, rather constant rates of information uptake across the dual-task condition should lead to a low probability of producing extreme deviations from the mean also during the bootstrapping process that equals that of the standard, single task, condition. Note also that the same arguments apply to the estimation of the whole set of parameters (i.e., also to *t*0 and *K* estimates).

### Calculation of dual task costs

To normalise the dual-task costs (see Boisgontier et al., 2013), the following formula was used when an increase in the metric was indicative of a dual-task cost (such as in the *t*0 parameter): DTC = [(DT − ST)/ST] × 100; when a decrease in the metric indicated a dual-task cost (as for the *C* and *K* parameters), then DTC = [(ST − DT)/ST] × 100 was used, instead (whereby DTC = dual-task costs, ST = single task performance, and DT = dual-task performance).

### Apparatus

To minimise distractions, the tests were administered in a dimly lit- and sound-attenuated room. The entire experiment was run on a Fujitsu Lifebook E series laptop, with a separate numeric keyboard used for the tapping task. However, for the presentation of stimuli, an ASUS VG248 17 inch monitor with a refresh rate of 100 Hz, a resolution of 1024 × 768 pixels. To ensure a viewing distance of 60 cms, both the seat on which the participant sat as well as the table on which the screen was placed were not moveable. Furthermore, the distance between the participant and the screen was demarcated with tape.

## Results

### Tapping results

Tapping performance in the single task was consistently high, with an average accuracy of 97.9% (SD = 3.9). There was no significant decline in the accuracy with which participants were able to complete the tapping task in the dual-task condition, although a tendency was found (*t* (23) = 1.41, *p* = .09). Tapping accuracy dropped to 96.7% (SD = 3.2) under dual-task conditions. Based on the above-mentioned formulas for normalising dual-task costs across participants, there was an average dual-task cost of 1.3% (SD = 4.5) in the dual-task condition.

### Whole report results

**Fig. 2:**
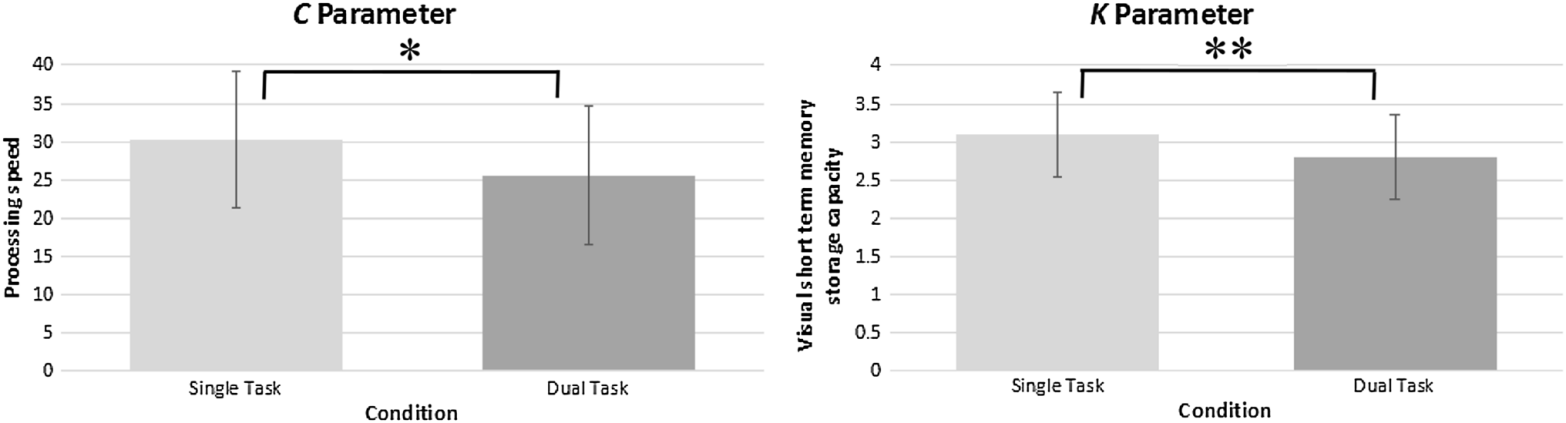
Single-task and dual-task results for parameter visual processing speed *C* and visual short-term memory storage capacity *K* respectively

**Fig. 3:**
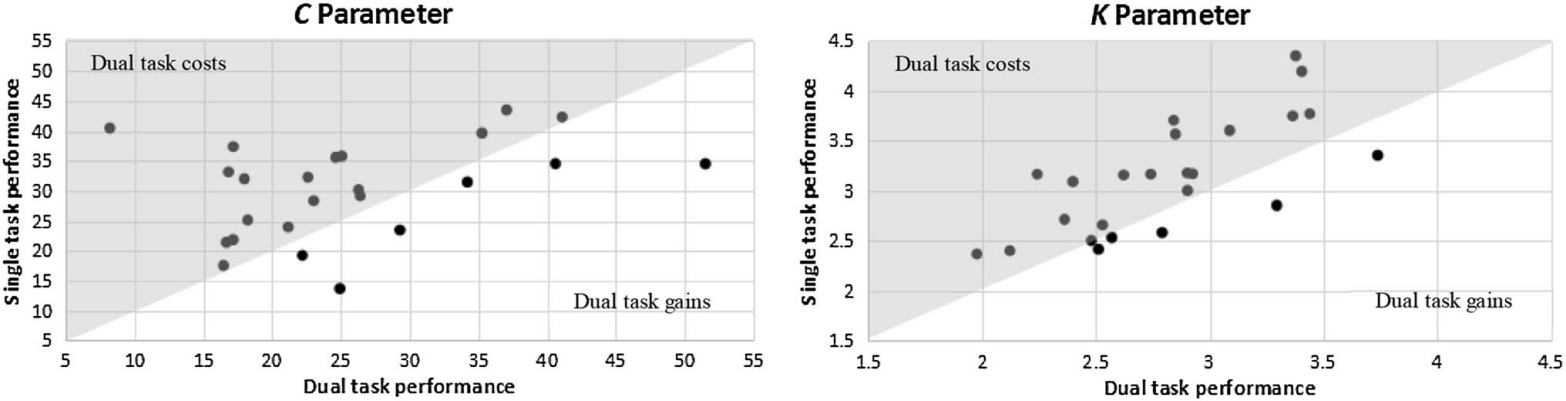
Individual dual-task costs in visual processing speed *C* and visual short-term memory storage capacity *K*

Accuracy of letter report as a function of effective exposure duration was modelled for each participant and each experiment condition by a TVA-based function that represented the maximum-likelihood fit to the data (Dyrholm et al., 2011; Kyllingsbæk, 2006). As can be seen in Fig. 2 below, in the single-task condition, participants had an average *C* value of 30.3 elements per second (SD = 8.1), whilst in the dual-task scenario, this parameter dropped to an average of 25.6 elements per second (SD = 10.0). A one-sided t test showed this difference to be significant (*t* (23) = 2.24, *p* = .02, *d* = 0.52). For VSTM storage capacity, participants had an average *K* parameter of 3.1 elements in the single task (SD = 0.6), and a mean *K* of 2.8 elements in the dual-task condition (SD = 0.5). A one-sided t test indicated this as a significant decline (*t* (23) = 4.07, *p* < .001, *d* = 0.63). Normalised dual-task costs in processing speed and VSTM storage capacity were also calculated, revealing an average cost of *M* = 11.6% (SD = 33.9) for the *C* parameter, and *M* = 9.47% (SD = 11.6) for the *K* parameter. As can be seen from Fig. 3, a decline in the *C* parameter occurred in 18, and a decrease in the *K* parameter in 19 of the 24 participants. In this figure, dual-task performance is plotted against performance in the single task. Thus, all data points falling within the gray triangle represent dual-task costs, whilst those falling within the white triangle represent a dual-task gain.

The parameter *t0*, or perceptual threshold, i.e., the minimum exposure duration at which participants start to process stimuli, was 17.4 ms (SD = 11.9) in the single task, whilst in the dual-task condition, the *t0* was 15.5 ms (SD = 10.1). This difference between the single-task- and dual-task conditions was not significant (*t*(23) = 1.14, *p* = .13).

Goodness-of-fit measures revealed that there was a close correspondence between the empirical mean scores in the different whole report conditions and the values that would be predicted based on the TVA parameter estimates. Average squared Pearson product-moment correlation coefficients of *R* ^2^ = 0.98 (SD = 0.02) in the single task and *R* ^2^ = 0.97 (SD = 0.03) in the dual task clearly indicated that, in both conditions, most of the variance in the empirical data was explained by the TVA model.

**Fig. 4:**
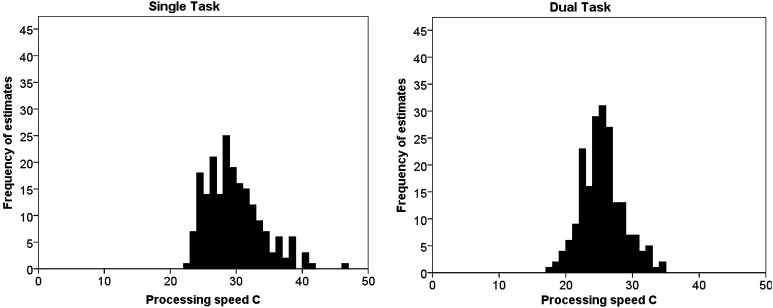
Distribution of a representative participant’s estimates for parameter visual processing speed *C* as obtained by bootstrapping

Resampling each original dataset with 200 bootstrapping iterations did not indicate any tendency for higher standard deviations for the resulting bootstrapping estimates of parameter processing speed *C* (single task: *M* = 5.29, SD = 2.38; dual task: *M* = 3.97, SD = 2.45) or VSTM storage capacity *K* (single task:  *M* = 0.12, SD = 0.04, dual task: *M* = 0.12, SD = 0.04). Figure 4 shows the distribution of bootstrapping estimates for parameter *C*, separately for the single- and the dual-task condition for a representative participant (whose estimates most closely resembled the mean group estimates in the single-task- and the dual-task conditions). Thus, there is no indication of increased variability in the bootstrapping estimates.

## Discussion

In this study, we combined a concurrent motor task, in the form of a repetitive finger tapping, with a visual task assessing the efficiency of visual information uptake. Based on TVA (Bundesen, 1990), parameter estimates were derived, both under single- and dual-task conditions, that reflected distinct components of visual processing capacity; that is, the perceptual threshold, the speed of visual processing, and the storage capacity of VSTM. Additionally, goodness-of-fit values were obtained for each condition to check whether parameters were validly estimated under both single- and dual-task conditions. Moreover, by applying a bootstrapping procedure, quantitative estimates of the reliability of the parameter estimates in each condition were obtained to test for possibly increased fluctuation of visual attentional performance in dual-task compared to single-task conditions.

Our results showed that concurrent tapping affected visual processing in a significant way. Both the speed of visual processing, and VSTM storage capacity declined under dual-task- compared to single-task conditions. In contrast, the perceptual threshold remained unaffected. These results suggest that a concurrent motor task taps attentional aspects of visual-processing capacity. Participants seem to process information at a lower rate and also to store less pieces of information in VSTM, but are not less sensitive for stimulus registration at minimal exposure durations.

The effect on processing capacity is remarkable when considering the fact that the tapping task was performed on a very high level, with more than 96% accuracy, under both single- and dual-task conditions. Obviously, then, tapping was not a very demanding task and subjects were readily able to keep motor performance in the dual-task condition on a level comparable to the single-task condition. Nevertheless, this rather easy task with only a minor cognitive demand was sufficient to significantly reduce efficiency of visual information uptake in participants at middle to higher age.

The analysis of goodness-of-fit values for the single- and dual-task conditions indicated that a very high variance of the empirical data was explained by the TVA parameter model estimates in both conditions. Moreover, bootstrapping analyses of the parameter estimates showed that the robustness of these estimates was comparable between single- and dual-task conditions. These results clearly do not suggest that the dual-task condition created a higher trial-to-trial variability in the way the participants approached the task. Instead, they support the assumption of the TVA-based fitting that relatively constant parameters underlie whole report performance of a given individual—also across the entire duration of the dual task.

These data are appealing for two reasons. First, they suggest that performing a concurrent motor task relies on attentional resources that are necessary for visual information uptake. Second, they are compatible with a capacity sharing account of motor-cognitive dual-tasking and justify the assumption that both tasks share a common central resource. Given the very short, near-threshold, exposure durations that are most critical for estimating visual processing speed *C*, these results would be difficult to reconcile with an attention switching account. Contrary to the prediction made by a switching account of dual tasking, there was no evidence of a time-based trade-off in processing the visual task under dual-task conditions, such that participants would switch between a state of paying attention (with a “normal” processing rate at the level of the single task), and a state of not paying attention to the display (with a rate of processing approaching 0). Such behaviour would be reflected in both a violation of the TVA model, giving rise to a decline in the goodness-of-fit, and in an increase of the variability of the bootstrapping estimates. Our analyses showed that this was not the case.

Of course, time-sharing accounts cannot be completely ruled out on the basis of our present findings. After all, there are lots of ways for costs of more difficult or higher-demand central processing to influence the time course of other processes (e.g., costs of switching between monitoring different tasks relative to task difficulty). Therefore, additional studies with experimental settings tailored to investigate this issue in more detail would be required. For example, combining TVA-based whole report with a “classical” psychological refractory period (PRP) paradigm could allow for more fine-grained temporal distinctions.

Our results also render another explanation for our data rather unlikely, namely that participants visually monitored the tapping device in the dual-task condition. The consistency with respect to both model fitting and bootstrapping estimates across single- and dual-task conditions speaks against such an assumption. Arguably, as participants would need to shift not only eye fixation but also turn their heads towards the tapping device, this should result in a marked change of visual threshold estimates (whereby trials with low-exposure duration in particular would be affected) and in reduced parameter robustness in general. Taken together, the high comparability between single- and dual-task conditions with respect to goodness-of-fit and bootstrapping estimates is in line with a resource sharing account predicting qualitatively similar but quantitatively less efficient visual processing in the dual compared to the single task.

Within the framework of TVA, parameter *C* reflects the amount of attentional capacity that can be allocated to the processing of objects in the visual field (Bundesen, 1990; Bundesen et al., 2015). Accordingly, a reduction of *C* would indicate that the amount of attentional capacity is decreased by the presence of a concurrent motor task. A plausible explanation would be that the motor task receives attentional weighting which leaves less attentional capacity available for visual processing. In other words, the concurrent motor task acts as sort of a distractor receiving attentional capacity. Two conclusions can be drawn from such an assumption. First, the decrease of processing speed assessed by the whole report task can be regarded as a quantification of the amount of attentional capacity that is used by the concurrent motor task. Second, due to the non-visual nature of the motor task, this suggests that central attention rather than visual attentional capacity is shared between the concurrent tasks. That is, the attentional capacity as conceptualized by TVA reflects, at least to some degree, central attentional resources instead of purely visual processing capacity. This has already been suggested by clinical studies in which processing speed has been associated with global cognitive ability (Bublak et al., 2011), or with a non-visual task reflecting central attentional capacity (Kluckow, Rehbein, Schwab, Witte, & Bublak, 2016). Note, however, that this is the first study to suggest a relationship between TVA-based visual processing speed and central attentional capacity in healthy subjects. While Poth et al. (2014) also found a reduction of processing speed under the influence of a concurrent visual task, this interference could be interpreted as a competition of visual attentional resources. Nevertheless, it must also be noted that both tasks involve a spatial component insofar as the TVA task utilises six stimuli spread out across the visual field, whilst the tapping task relies on the learning of a sequence which is spatially organised. Thus, it is also possible that rather than drawing on a general central attentional capacity, the tasks more specifically tap into a form of spatial attention. However, it is not possible to distinguish the degree to which the attentional changes found in this paper are reflective of either spatial attention or a more general attentional capacity.

The *K* parameter reflects VSTM storage capacity in TVA, which represents object categorisations that are available for further processing. Essentially, and in accordance with the ECTVA framework of Logan and Gordon (2001), this is a stage of response selection, which results in naming of the letters in the case of whole report. In the presence of a concurrent motor task, response selection is made more demanding by the fact that not only do letters have to be named, but also that finger movements need to be selected. Here, executive control is necessary, and our results suggest that this stage is also characterized by resource sharing. A possible explanation could be that when more representations have to be maintained in parallel in a passive store such as VSTM, the reliability of these representations is reduced, owing to decay or interference (see e.g., Jonides et al., 2008), and response selection is rendered more difficult.

A limitation of our study is that our investigation involved subjects of middle to higher age. Therefore, the results need first to be replicated in younger subjects, before their applicability can be reliably evaluated. However, our results can provide a first step towards a deeper understanding why motor-cognitive dual-task effects seem to be especially pronounced under concurrent visual processing demands in the elderly (Boisgontier et al., 2013). Furthermore, they set a valuable framework for neuropsychological studies in patients with lesions in brain regions relevant for cognitive-motor functions, which are currently underway.
